# Integrated physiological and transcriptomic data revealed the cold-resistant mechanisms in reproductive organs of the ‘Jinguang’ pear cultivar

**DOI:** 10.3389/fpls.2024.1501774

**Published:** 2025-02-03

**Authors:** Mengying Sun, Shun Lin, Zezhao Zhao, Weizhen Guo, Min Jiang, Ying Li, Jun Zhang, Jingxian Zhao, Minsheng Yang

**Affiliations:** ^1^ Hebei Agricultural University, Baoding, Hebei, China; ^2^ Hebei Key Laboratory for Tree Genetic Resources and Forest Protection, Baoding, Hebei, China; ^3^ Hebei Academic of Forestry and Grassland, Shijiazhuang, Hebei, China

**Keywords:** low-temperature stress, pear, flower organs, young fruit, flavonoid biosynthesis

## Abstract

The *Pyrus* spp. (pears) are crucial for the fruit industry; however, low spring temperatures can cause frost damage to their reproductive organs, which poses challenges to the final yields. In this study, we evaluated the response of the flowers and young fruits of the ‘Jinguang’ pear cultivar to low temperatures from integrated phenotypic, physiological, and molecular approaches. We found that the flowers were less sensitive to low temperatures than the young fruits, of which their over-cooling points were −5.6°C and −5.0°C, respectively. Transcriptomic data showed that the differentially expressed genes from flowers and young fruits compared to the control conditions were primarily involved in the biosynthesis of flavonoids, phenylalanine, and tyrosine. Further weighted gene co-expression network analysis uncovered the core transcription factors that may be potentially involved in the pear cold resistance, including *MYB20*, *WRKY53*, and *WRKY30*. Our findings provide valuable insights and candidate gene resources for further exploration of the molecular mechanisms underlying cold resistance in pear trees.

## 1 Introduction

Due to the global climate change in recent years, extreme weather events frequently occur (Wang et al., 2023). During their whole life cycle, plants are often faced with various biotic (e.g., diseases and pests) or abiotic (e.g., drought, salinization, and extreme temperature) stress conditions (Gechev and Petrov, 2020). These adverse factors affect and/or restrict the growth and development of plants to a large extent (Furlong et al., 2013). The impact of low temperatures on the growth and development of plants is significantly pronounced. The degree of damage caused by low-temperature stress depends largely on the severity of the chill, the duration of exposure, and the specific developmental phase of plants (Xiao et al., 2022). The impacts of low-temperature stress on plants are typically categorized into frost damage, chilling injury, and frost damage, based on distinct temperature conditions, seasons of occurrence, and regions affected (Mccully et al., 2004). These forms of low-temperature stress can result in mechanical cell damage in plants, hence diminishing the quality and the final yield of both food and fruit crops (Lukatkin, 2005).

Plants have evolved a series of adaptive mechanisms to counteract low-temperature stress, encompassing antioxidant enzyme systems, non-enzymatic defense systems, and secondary metabolic defense mechanisms (Jin et al., 2011). These mechanisms integrally mitigate the accumulation of reactive oxygen species (ROS) and alleviate the intercellular oxidative damage induced by the low temperatures. The antioxidant enzyme system comprises a set of enzymes, including superoxide dismutase (SOD), peroxidase (POD), and catalase (CAT) (Yao et al., 2018). SOD can convert superoxide anions (O_2_•−) into oxygen (O_2_) and hydrogen peroxide (H_2_O_2_). POD and CAT further reduce H_2_O_2_ into H_2_O, preventing H_2_O_2_ from accumulating to harmful levels for the plant cell (Fluhr, 1995). The non-enzymatic defense system primarily comprises various osmoregulators, such as soluble sugars (SS) and proline (Pro). These compounds enable them to maintain and balance the osmotic pressure across the cell membrane, thus mitigating the damage to the plasma membrane induced by low-temperature stress (Kovinich et al., 2015). Secondary metabolites are broadly classified into three major groups: terpenes, phenolic compounds, and alkaloids (Li et al., 2023). Flavonoids, a subset of phenolic compounds, play a significant antioxidant role in plants. The phenolic hydroxyl groups in their molecular structure serve as hydrogen donors, capable of directly reducing ROS into stable and less harmful molecules, thus helping to alleviate oxidative stress and protect cells from damage (Treml and Mejkal, 2016). Additionally, low temperatures can significantly induce the expression of resistance genes that encode transcriptional factors in plants, directly or indirectly modulating the downstream molecular pathways, finally making plants as resistant as possible to low temperatures (Zhang et al., 2024). For instance, the increased expression of the *Arabidopsis thaliana CBF1* gene, encoding an AP2/ERF type transcriptional activator, directly binds to the CRT/DRE sequence, inducing *COR* gene expression and thus enhancing the freezing tolerance in non-acclimated *Arabidopsis* plants (Jagllo-Ottosen et al., 1998).

Pear trees (*Pyrus* spp.) are deciduous trees of the family Rosaceae (Wang et al., 2014). Pear flowers, renowned for their ornamental value, are frequently incorporated into landscapes (Reiland and Slavin, 2015). The pear fruit is known for its juicy, sweet, and unique flavor, along with being rich in vitamins and minerals (Singh et al., 2024). Additionally, pear fruits also have medicinal value, which helps relieve coughs, clear phlegm, lower blood pressure, and strengthen the immune system (Ma et al., 2024). Flowering and fruiting phases are crucial for the reproduction of the fruit crops (Djaman et al., 2021). As for pear trees, their flowers and fruits are particularly susceptible to damage from prolonged nighttime low temperatures, leading to the browning and dropping of the flowers and the wilting of the young fruits, which adversely affect pear yields and quality (Wang et al., 2015).

Advancements have been made in low-temperature tolerance mechanisms for fruit trees to low-temperature exposure in recent years. For example, existing studies have already detailed and examined the physiological and morphological characterizations in apricots, peaches, plums, oranges, and apples during flowering and fruiting development stages exposure to low temperatures (Liu et al., 2003; Liu et al., 2020; Wang et al., 2015; Yang et al., 2023). For pears, the majority of research has focused on the effects of exogenous glycine betaine, ATP treatment, and 1-methylcyclopropene in preventing low temperature-induced browning in its fruit peel. However, the critical thresholds of frost resistance for young fruits and flower organs of pear trees, as well as its molecular mechanisms underlying low-temperature resistance, remain largely a mystery (Luo et al., 2022; Tao et al., 2019; Zhang et al., 2018).

In this study, we aimed to deepen the understanding of low-temperature resistance mechanisms in pear organs across different phenological periods. We assessed the supercooling points and physiological parameters of young fruits and flower organs under chilling treatments with different temperatures. Applying comparative transcriptomic approaches and using advanced weighted gene co-expression network analysis (WGCNA), we screened key genes, including *MYB20*, *WRKY53*, and *PDLP6*, from the massive data, which were suspected to play a significant regulatory role in responses to low-temperature induction in pear. Our results lay a theoretical foundation for further understanding of the mechanisms by which the reproductive organs of pears cope with low temperatures. These data presented in our research are also contributing precious gene resources for the development of cold-resistant germplasms in the future.

## 2 Materials and methods

### 2.1 Study site and plant materials

The study site is located in the Pear Germplasm Resources Nursery of the Hebei Forestry and Grassland Research Institute (41°35′N, 114°28′E), China. The site has a temperate continental monsoon climate, with an annual average humidity of 65%. Temperatures are lowest between December and February; however, frost damage may still occur in March and April.

The ‘Jinguang’ cultivar is a cross between the ‘Huangjin’ and ‘Huangguan’ pear cultivars. It is a new pear variety with middle to early maturity, high and stable yield, and strong cold resistance (Guo et al., 2022). This study was conducted in 2021. When the young pear fruit had grown to 2–3 cm, three pear trees with healthy growth potential, similar shape, and consistent cultivation and management methods were selected. A total of 60 branches with consistent thickness and length (50–80 cm) and bearing young fruit from the same height on each selected plant were randomly collected. These branches were transported to the laboratory, and the young fruits were picked and divided into five groups for subsequent low-temperature treatment. The fruits were placed in a frost simulator (model: MSZ-2F) for low-temperature treatment at 2°C (treatment M1), 0°C (M2), −2°C (M3), or −4°C (M4). Control fruits were kept at 18°C (CK). The frost simulator was pre-cooled from room temperature to 10°C, and then the temperature was reduced to the desired temperature at a rate of 2°C/h and maintained for 4 h. After treatment, the samples were wrapped in foil, frozen in liquid nitrogen, and stored at −80°C for sequencing and physiological analyses.

### 2.2 Low-temperature treatment conditions

The flowers and young fruits were placed in the MSZ-2F frost box at 0°C, 1°C, 2°C, 3°C, 4°C, 5°C, 6°C, and 7°C. The PT-100 thermocouple temperature sensor was placed in the position to be measured. The temperature sensor was connected to the FrosTem40 data acquisition system and microcomputer and scanned once every 10 s. The surface temperature of flowers and young fruits was recorded continuously at a rate of 1°C/0.5h, 4°C for 30 min, and maintained for 2 h at −7°C. The fruits were then restored to room temperature at a rate of 4°C/h and used for the determination of the over-cooling points, with three biological replicates per treatment. Relative conductivity was calculated by measuring the initial and final conductivity using a conductivity meter (DDS-303A, Nanbei Instrument, Ltd., Shanghai, China) (Nayyar, 2003). SS and Pro contents were determined using a kit (Gris Biotechnology Co., Ltd., Suzhou, China). Soluble protein (SP) content was determined using the Coomassie Brilliant Blue G-250 method (Shukla et al., 2012), SOD activity was determined using the nitroblue tetrazolium photoreduction method, POD activity was determined using the guaiacol method, CAT activity was determined using the hydrogen peroxide method (Jiang et al., 2022; Wang et al., 2024), and malondialdehyde (MDA) content was determined using the thiobarbituric acid method (He et al., 2015). All experiments and assays were performed in triplicate. Some physiological data for flower organs were obtained from previous studies performed by our research group for comparison (Lin et al., 2023).

### 2.3 Transcriptome sequencing analysis

Transcriptome samples from ‘Jinguang’ pear flower organs and young fruit exposed to different temperatures were sequenced, with three biological replicates per group. Sequencing was performed by Kidio Biotechnology Co., Ltd. (Guangzhou, China). Total RNA was extracted from each sample using TRIzol reagent, and RNA integrity was accurately detected using a bioanalyzer (Agilent 2100, Agilent Technologies, Santa Clara, CA, USA). Quality control of the original transcriptome sequencing data was performed using the fastp pre-processing tool to obtain high-quality reads. The reference genome was then aligned using HISAT2, and the results were used to reconstruct the transcripts with Stringtie; the expression levels of all genes in each sample were calculated using the RSEM tool. Based on the differences between groups, genes with a false discovery rate <0.05 and |log_2_(fold change)| > 1 were defined as differentially expressed genes (DEGs) and subjected to Gene Ontology (GO) and Kyoto Encyclopedia of Genes and Genomes (KEGG) enrichment analyses, using a threshold of Q ≤ 0.05.

Key regulatory genes in ‘Jinguang’ pear fruit were identified through WGCNA, conducted using the Omishare online tool, with a soft threshold of β = 7. According to the clustering relationship among genes, gene modules were used to obtain a hierarchical clustering tree; an association analysis between the module eigenvalues and specific shape data was performed to identify the most relevant modules and to search for other genes associated with that shape, where the gene significance (GS) value reflects the correlation between each gene and the trait and the module membership (MM) value is effectively the correlation coefficient between the expressed gene and the characteristic value of the module. Thus, a stronger correlation indicates greater significance of the module for this shape. Genes with GS > 0.7 and MM > 0.8 were defined as core genes and used to construct a regulatory relationship network map of the core genes using the Cytoscape software.

### 2.4 Quantitative reverse transcription–polymerase chain reaction

The expression levels of genes identified by RNA sequencing (RNA-seq) were verified by reverse transcription–polymerase chain reaction (qRT-PCR) of the six DEGs in the core module, using the tubulin gene as a reference gene. Primers were designed using Primer Premier 6.0; their sequences are listed in **Supplementary Table S1**. A first-strand synthesis kit (SENO BioTech, Zhangjiakou, China) was used to synthesize the cDNA. qRT-PCR was performed using the SYBR Green 2X qPCR kit. These experiments were repeated three times, and the relative expression of target genes was calculated using the 2^−ΔΔCt^ method.

### 2.5 Statistical analysis

Physiological index data were collated using Excel software (ver. 2016, Microsoft Corp., Redmond, WA, USA) and statistically analyzed using SPSS ver. 27.0 (IBM Corp., Armonk, NY, USA). Duncan’s test and analysis of variance (ANOVA) were performed to test for differences between samples. The results were plotted using Origin ver. 2021 (OriginLab Corp., Armonk, NY, USA) and was further optimized using Photoshop ver. 2023 (Adobe, San Jose, CA, USA).

## 3 Results

### 3.1 Phenotypic characteristics and subcooling point of fruit following cold treatment

**Figure 1:**
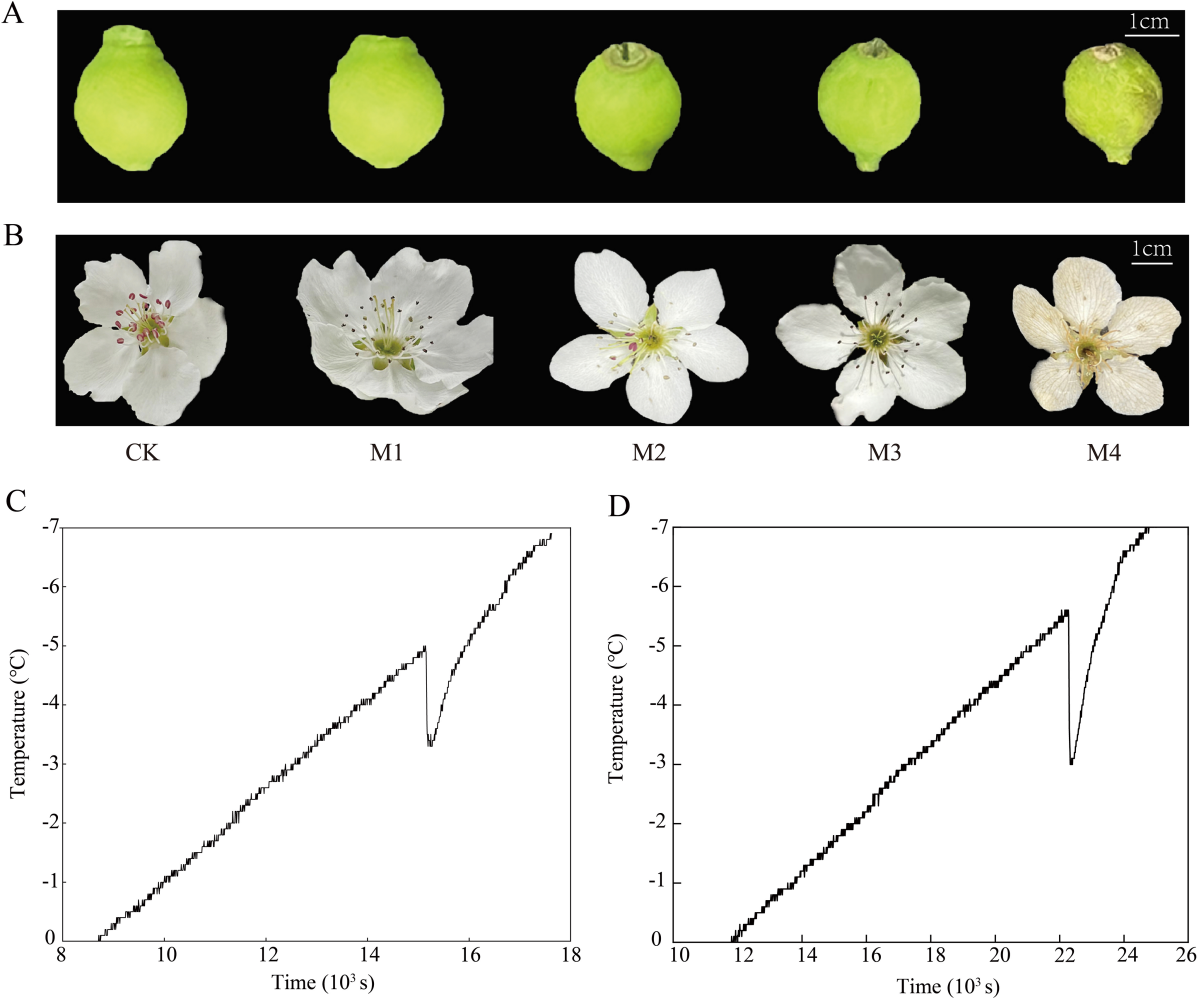
Phenotypic changes **(A, B)** and over-cooling points **(C, D)** of ‘Jinguang’ pears under low-temperature treatments. **(A, B)** Phenotypic changes of the young fruit and the flower organs, respectively. **(C, D)** Over-cooling points of the young fruit and the flower organs, respectively. CK, M1, M2, M3, and M4 in panels **(A, B)**: temperatures set at 18°C, 0°C, 2°C, −2°C, and −4°C, respectively.

Tissue discoloration and water-loss wilting are the most intuitive manifestations of young fruit and flower organs subjected to low temperatures. The damage to young fruit and flower organs of ‘Jinguang’ was investigated and photographed under different temperatures. The results showed that the phenotypes of young fruits did not change at the temperatures of CK, M1, and M2 but wilted slightly at the temperatures of M3; the color of young fruits began to turn brown and yellow; serious wilting and water loss occurred at the temperatures of M4 (**Figure 1A**) At CK, M1, M2, and M3, the phenotypic changes of flower organs were not obvious, while at M4, browning occurred (**Figure 1B**). The supercooling points of ‘Jinguang’ young fruit and flower were −5.0°C and −5.7°C, respectively, and the supercooling points of young fruit were 0.7°C higher than those of flower organs (**Figures 1C, D**). It is generally considered that the lower the supercooling point, the higher the cold resistance ability of the plant tissues. Thus, the flowers were suspected to possess a more resistant ability to chilling stress than the young fruits, which were next explored.

### 3.2 Comparison of physiological indicators of fruit and flower organs following cold treatment

**Figure 2:**
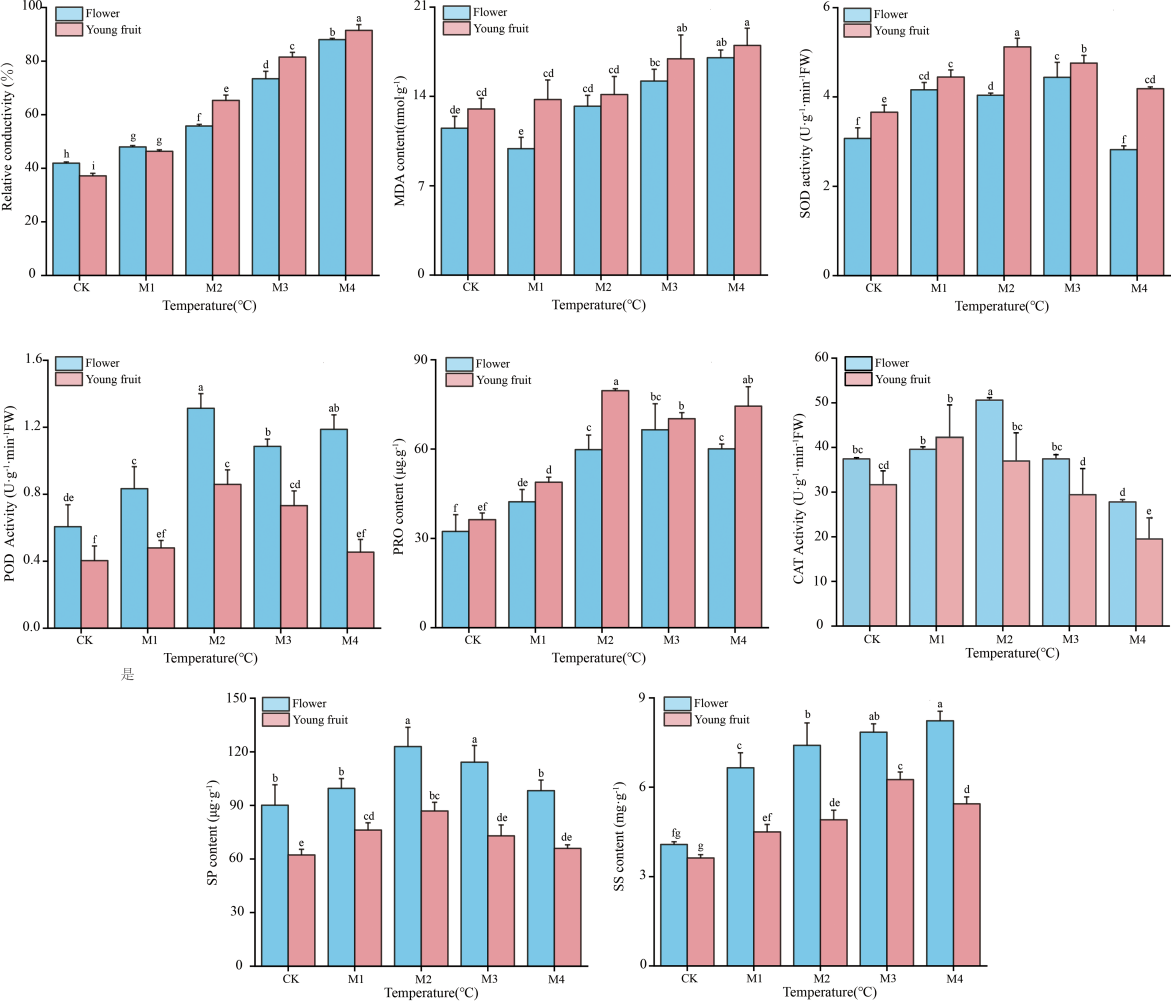
Physiological indicators of ‘Jinguang’ pears following different temperature treatments. The letters that differ completely show significant variations. CK, M1, M2, M3, and M4 in temperatures set at 18°C, 0°C, 2°C, −2°C, and −4°C, respectively.

Relative conductivity; MDA, SS, SP, and Pro contents; and SOD, POD, and CAT activities showed similar trends between fruit and flower organs (**Figure 2**). Peak SOD and CAT activities and peak SS and Pro contents were observed at temperatures 2.0°C higher in fruit than in flower organs. After these peaks were reached, the rate of decline was faster in fruit than in flower organs, indicating that young fruit were more sensitive to low temperatures. Flower organs had higher POD and CAT activities, higher SS and SP levels, lower MDA content, and lower relative conductivity than fruit at temperatures lower than the M1 treatment temperature. These results indicate that high levels of antioxidant enzyme activity, a high osmoregulatory capacity, and low lipid peroxide levels contribute to greater cold resistance in flower organs compared to young fruit.

### 3.3 Analysis of common differentially expressed genes in young fruits and flowers under low-temperature treatment

**Figure 3:**
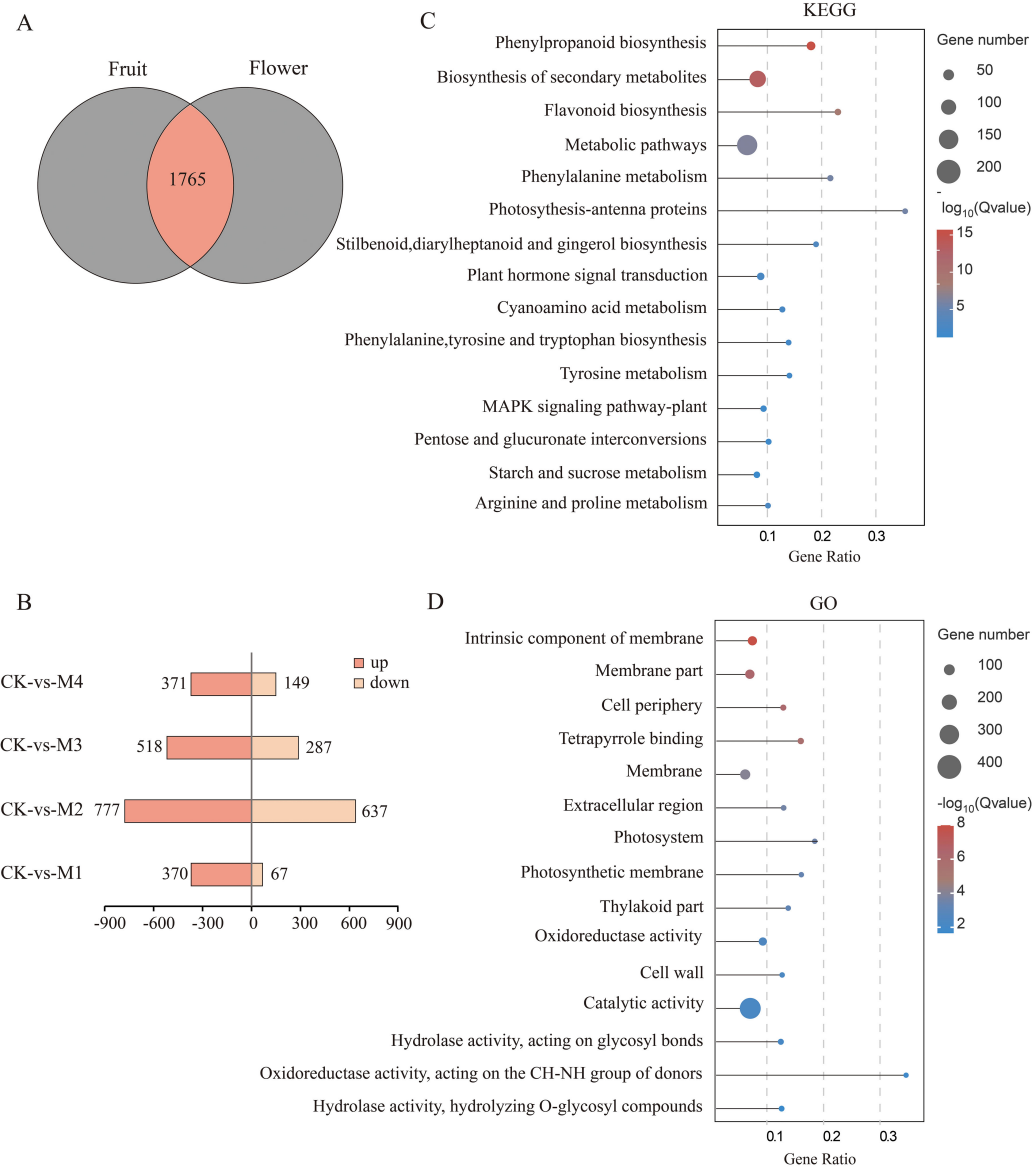
Analysis of differentially expressed genes (DEGs) shared between young fruits and flowers. **(A)** Venn diagram of DEGs. **(B)** Pairwise comparison of up- and downregulated DEGs. **(C, D)** Top 15 Kyoto Encyclopedia of Genes and Genomes (KEGG) and Gene Ontology (GO) significantly enriched pathway plots among the common DEGs of young fruit and flower organs (*p* < 0.05).

The number of DEGs shared by young fruits and flower organs was 1,756 (**Figure 3A**), and we mapped their up- and downregulation compared with CK (**Figure 3B**), revealing 437 DEGs in M1 (370 upregulated and 67 downregulated), 1,414 DEGs in M2 (777 upregulated and 637 downregulated), 805 DEGs in M3 (518 upregulated and 287 downregulated), and 520 DEGs in M4 (371 upregulated and 149 downregulated), representing in total 2,036 upregulated genes and 1,140 downregulated genes. The numbers of DEGs in each treatment decreased in the order M2 > M3> M1 > M4. The higher numbers of upregulated than downregulated genes indicated that pear fruit and flower organs resist cold by upregulating the expression of large numbers of genes. Our KEGG analysis showed that DEGs were involved in phenylpropanoid, secondary metabolite, flavonoid, stilbenoid, diarylheptanoid, gingerol, phenylalanine, tyrosine, and tryptophan biosynthesis; phenylalanine, starch, sucrose, arginine, and proline metabolism; and pentose and glucuronide interconversion pathways (**Figure 3C**). Our GO analysis results showed that the DEGs were involved in intrinsic components of the membrane; the photosystem and photosynthetic membrane; oxidoreductase, catalytic, and hydrolase activities; and hydrolyzing *O*-glycosyl compounds (**Figure 3D**).

### 3.4 Comparison of differentially expressed genes specific to young fruits and flowers under low-temperature treatment

**Figure 4:**
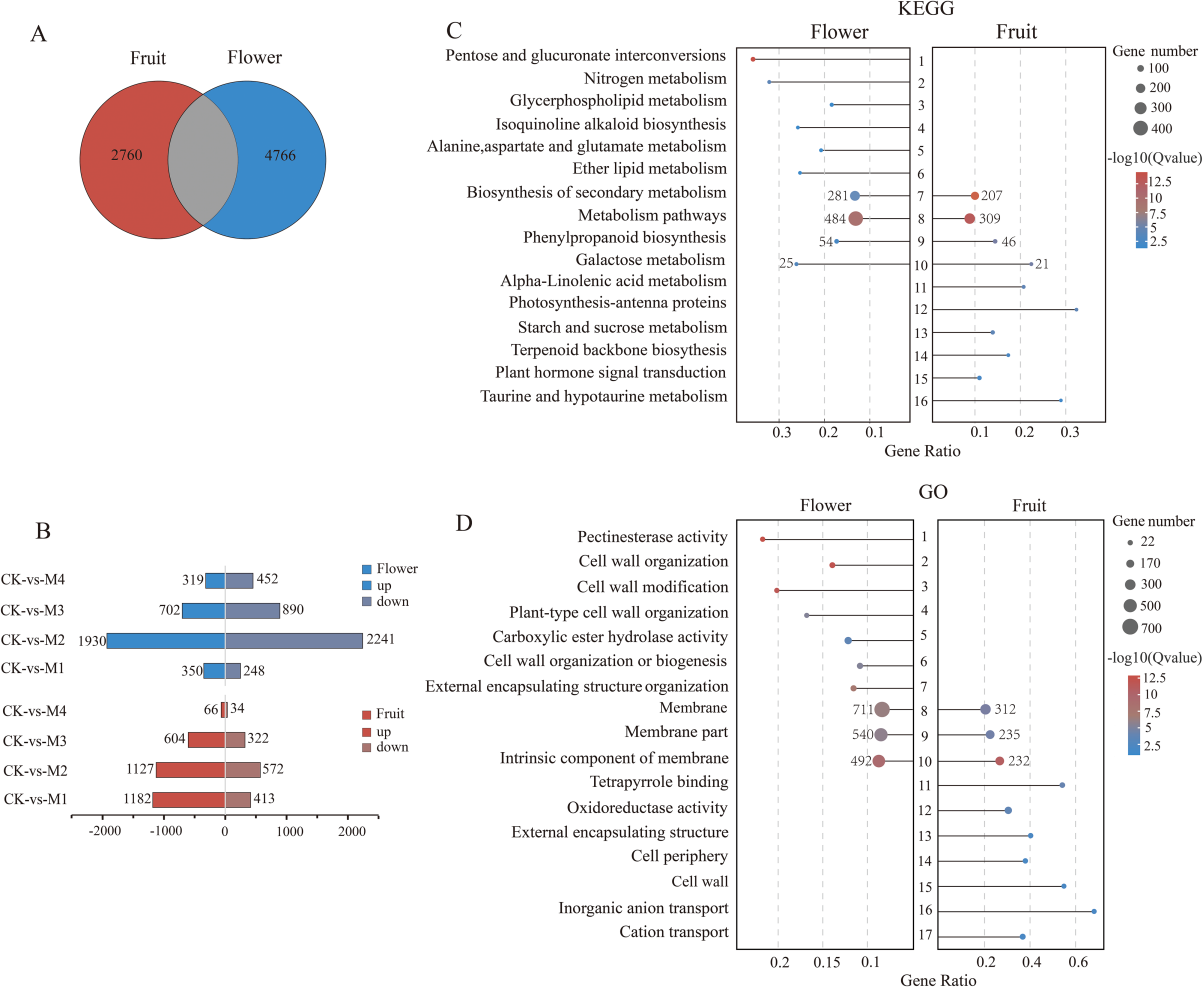
Differentially expressed genes (DEGs) unique to pear young fruit and flowers. **(A)** Venn diagram of DEGs. **(B)** Pairwise comparisons of up- and downregulation patterns of unique DEGs between pear fruit and flower organs. **(C, D)** The Kyoto Encyclopedia of Genes and Genomes (KEGG) and Gene Ontology (GO) pathways were significantly enriched in the top 10 young fruits and flowers (*p* < 0.05).

A comparison of DEGs between pear fruit and flower organs revealed 4,525 DEGs in fruit, of which 2,760 were unique, and 6,531 DEGs in flower organs, of which 4,766 were unique (**Figure 4A**). Both sets of unique DEGs were subjected to KEGG and GO analyses. Among fruit, 2,979 unique DEGs were upregulated and 1,341 were downregulated, most of which were in the M1 treatment group (**Figure 4B**). Among flower organs, 3,301 unique DEGs were upregulated and 3,831 were downregulated, most of which were in the M2 treatment group. KEGG analysis showed that DEGs unique to fruit or flower organs shared common pathways including secondary metabolite and phenylpropanoid biosynthesis and galactose and other metabolic pathways (**Figure 4C**). More of these DEGs were present in flower organs than in fruit, which may explain the stronger cold resistance of flowers.

The terpenoid backbone biosynthesis and taurine, hypotaurine, and alpha-linolenic acid metabolic pathways were unique to fruit, whereas the nitrogen, glycerophospholipid, alanine, aspartate, and glutamate metabolism pathways were unique to flower organs. GO analysis showed that intrinsic and other membrane components were enriched in both fruit and flower organs, whereas inorganic anion and cation transport were enriched only in fruit and pectin–esterase activity; cell wall modification, organization, and biogenesis, and carboxylic ester hydrolase activity were enriched only in flower organs (**Figure 4D**). These differential pathway enrichment patterns may indicate the main factors underlying differences in cold resistance mechanisms between pear fruit and flower organs.

### 3.5 Cluster heatmap of osmotic regulators and antioxidant enzymes following cold treatment

**Figure 5:**
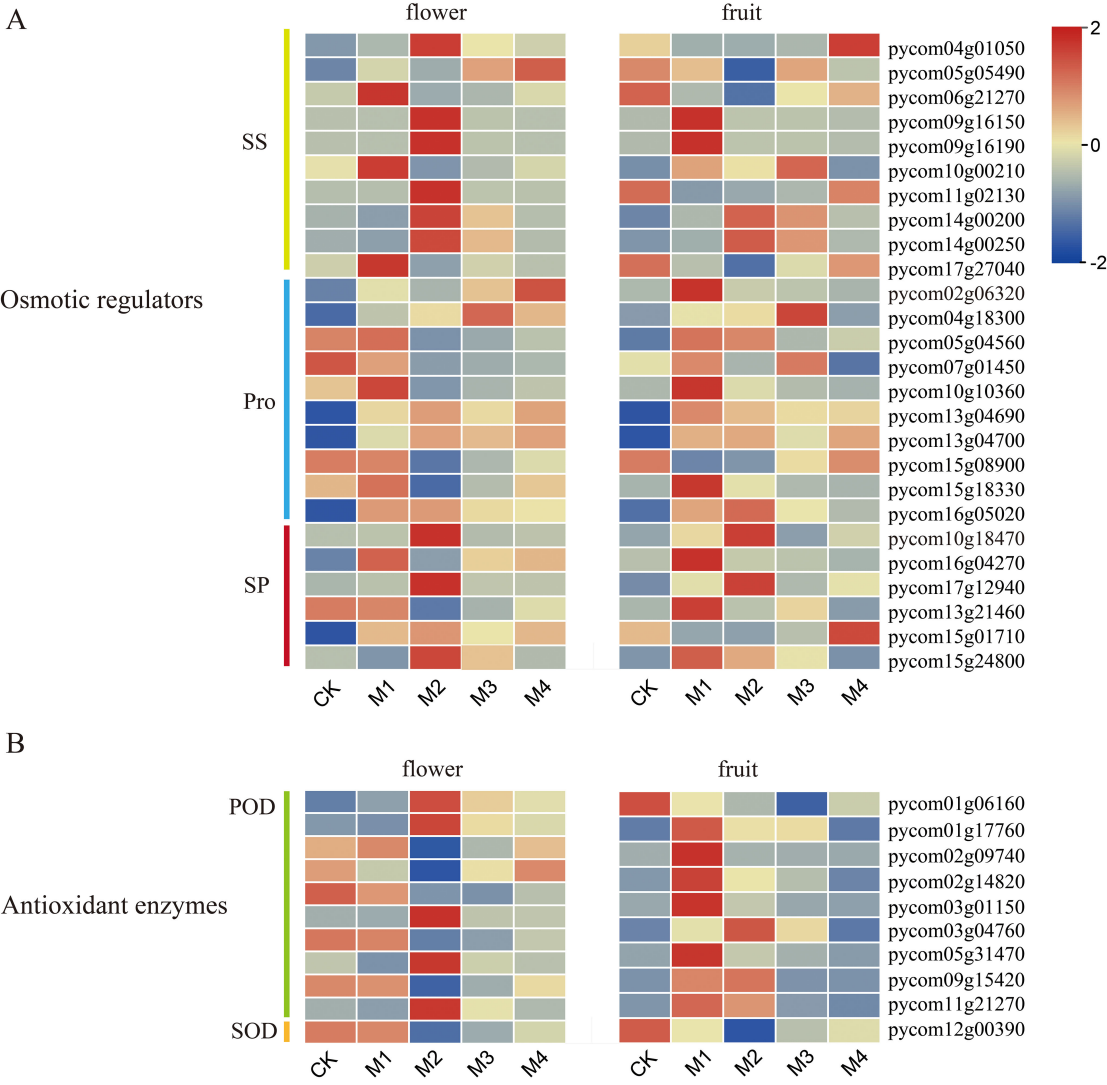
Heatmap of genes related to **(A)** osmoregulatory substances and **(B)** antioxidant enzymes in pear fruit and flowers.

DEGs shared by fruit and flower organs included those related to osmoregulatory substances and antioxidant enzymes (**Figure 5**). For example, among genes related to SS content, six genes in flower organs were significantly upregulated in the M2 group, and three were significantly upregulated in M1, whereas two genes in fruit were significantly upregulated in the M1 group and one was upregulated in M4. Among genes related to Pro content, six were upregulated and eight were significantly upregulated in flower organs and fruit, respectively, in the M1 group. Among the six genes related to SP content, three were significantly upregulated in flower organs in the M2 group, and three were significantly upregulated in fruit in the M1 group. Among genes related to POD activity, five were significantly upregulated in flower organs in the M2 group, and seven were significantly upregulated in fruit in M1. As the treatment temperature decreased, DEGs related to antioxidant enzymes and osmoregulatory substances were mainly upregulated, potentially alleviating damage to fruit and flower organs caused by cold stress by regulating the peroxidase system and osmoregulatory substances. Most genes associated with SS and SP contents and POD activity were upregulated at lower temperatures in flower organs than in fruit, which may explain the greater sensitivity of young fruit to low temperatures.

### 3.6 Common flavonoid biosynthesis-related pathways between fruit and flower organs

**Figure 6:**
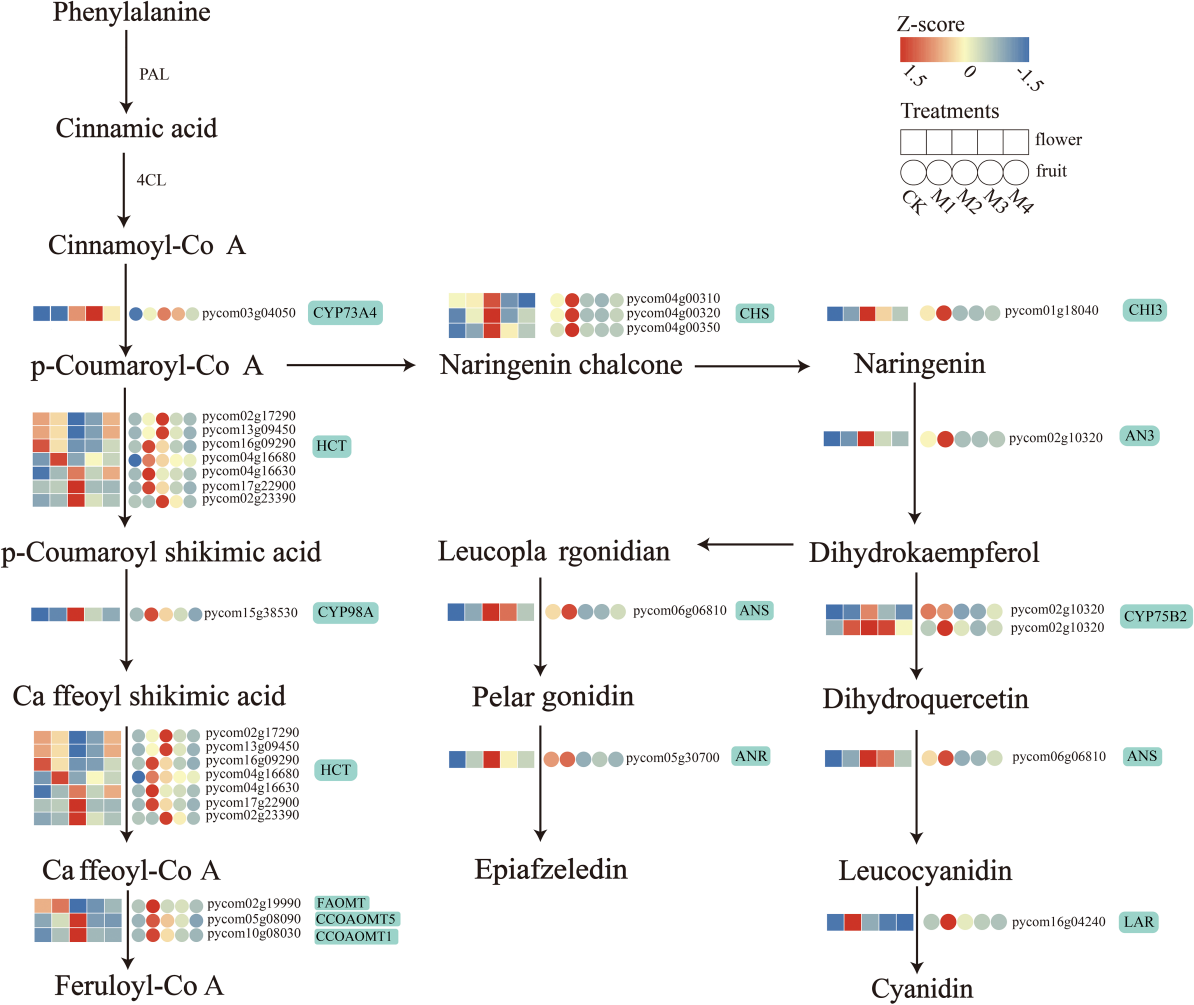
Biosynthetic pathway diagram for differentially expressed genes (DEGs) encoding flavonoids in both pear fruit and flower organs.

Several DEGs with pathway enrichment patterns shared by fruit and flower organs were involved in flavonoid biosynthesis. To further explore the effect of low-temperature treatment on flavonoid structural genes in fruit and flower organs, we visualized the transcript abundance of 22 of these DEGs (**Figure 6**). The DEGs included one gene encoding *trans*-cinnamate 4-monooxygenase (CYP73A4), seven genes encoding shikimate *O*-hydroxycinnamoyltransferase (*HCT*), one gene encoding 5-*O*-(4-coumaroyl)-d-quinate 3′-monooxygenase (*CYP98A2*), three genes encoding caffeoyl-CoA *O*-methyltransferase (*CCOAOMT1*, *CCOAOMT5*, and *FAOMT*), three genes encoding chalcone synthase (*PKS 5*, *CHS 2*, and *PKS 1*), one *ANS* gene, one CHI gene (*CHI3*), one naringenin 3-dioxygenase (*AN3*) gene, two genes encoding flavonoid 3′-monooxygenase (*CYP75B2*), one gene encoding ANR, and one gene encoding *LAR*. Among these, *CYP73A4* was significantly upregulated; its highest expression level in flower organs occurred in the M3 treatment, and that in fruit occurred in the M2 treatment. DEGs encoding *CHS*, *CHI3*, *ANS*, *CYP98A*, *ANR*, *CCOAOMT*, and *AN3* were upregulated; their highest expression levels in flower organs occurred in the M2 treatment, and those in the fruit occurred in the M1 treatment. All seven *HCT* genes were upregulated in fruit, and four genes were upregulated in flower organs, with *pycom02g17290* and *pycom13g09450* expressed at the lowest levels in floral organs in the M2 treatment and the highest levels in fruit. *LAR* genes in both fruit and flower organs were upregulated under cold treatment.

### 3.7 Co-expression networks of key genes in fruit exposed to cold stress

**Figure 7:**
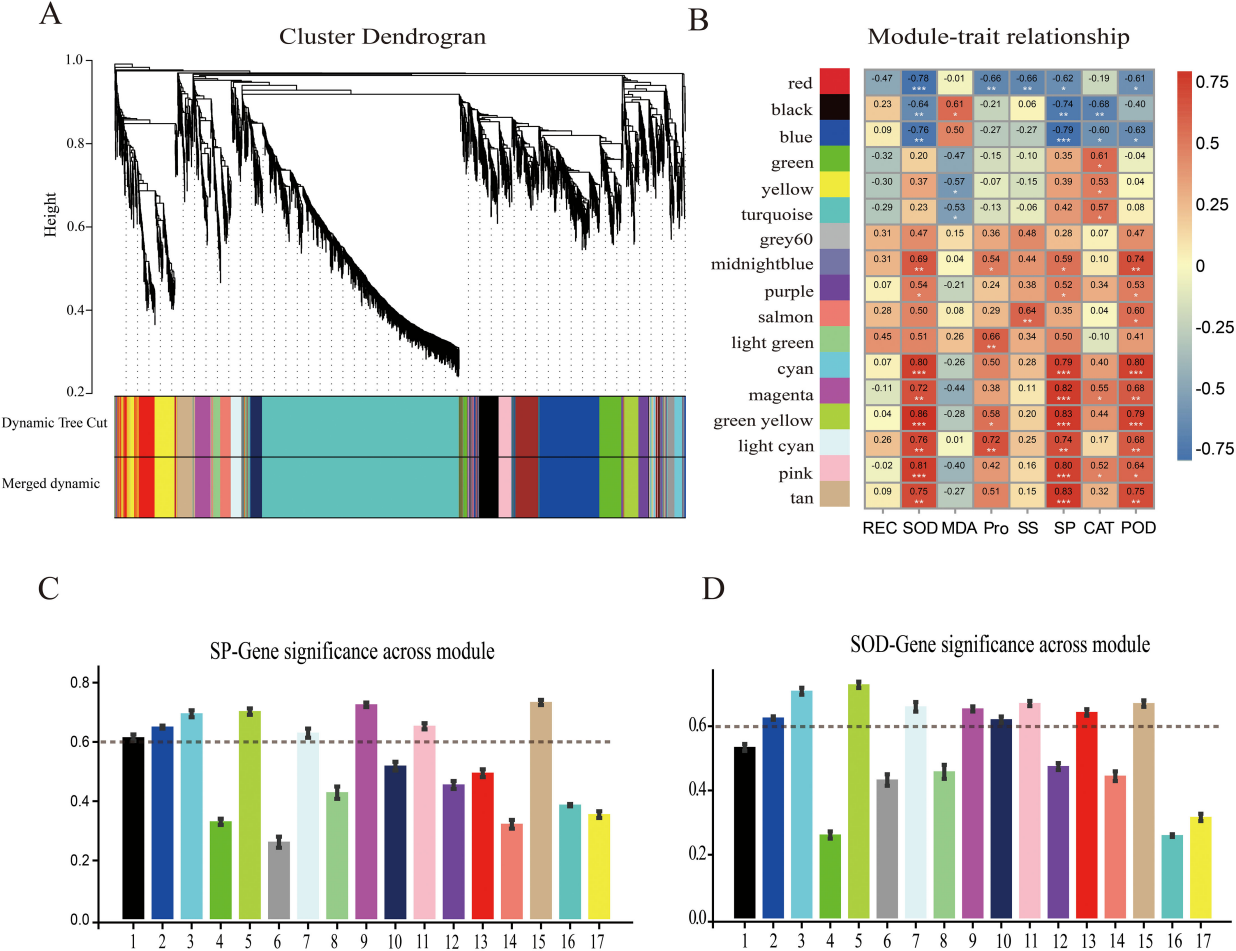
Weighted gene co-expression network analysis (WGCNA) and module–trait correlation analysis. **(A)** Hierarchical cluster tree of co-expression modules identified by dynamic cutting; the main branches constitute 17 modules. **(B)** Correlation analysis of physiological indices and modules; each column on the abscissa corresponds to one module. Numbers indicate correlation coefficients between physiological indicators and modules; asterisks indicate significant correlations (*p < 0.05, **p < 0.01, ***p < 0.001). Correlation analyses of WGCNA modules with **(C)** soluble protein (SP) content and **(D)** superoxide dismutase (SOD) content.

The gene regulatory network of ‘Jinguang’ pear materials under low-temperature stress and specific genes closely related to their physiological changes were further explored through WGCNA of 4,525 DEGs identified by RNA-seq. The weighted gene co-expression network was constructed based on a soft threshold power of β = 7. In the WGCNA, 17 modules were identified using the dynamic tree-cutting technique (**Figure 7A**), among which significantly positive correlations were determined between physiological indicators and the cyan, green-yellow, light cyan, pink, tan, and magenta modules, and significantly negative correlations were determined between physiological indicators and the red and black modules (**Figure 7B**). According to these correlations, a critical threshold of GS ≥ 0.6 was determined. Among modules with significant correlations, the cyan, green-yellow, and tan modules were significantly positively correlated with SP (**Figure 7C**), whereas the green-yellow, cyan, and light cyan modules were significantly positively correlated with SOD content (**Figure 7D**).

### 3.8 Analysis of key genes associated with SP accumulation

**Figure 8:**
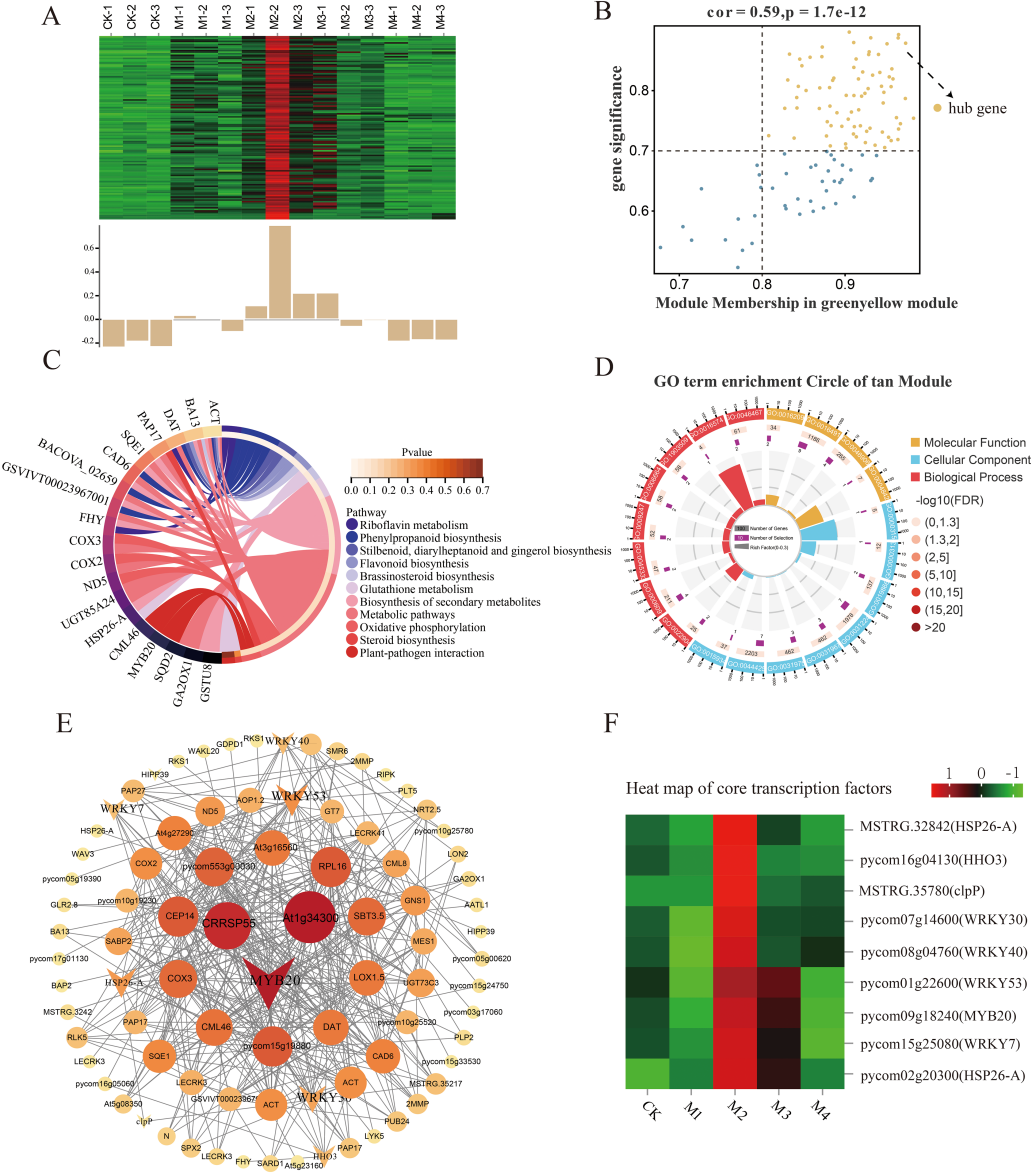
Co-expression network of the tan module. **(A)** Gene co-expression heatmap for the tan module (top) and characteristic gene expression for each sample (bottom). **(B)** Dot plot of central genes with high expression and connectivity between soluble protein (SP) content and the tan module; the horizontal axis indicates the correlation strength between each expressed gene and a module, and the vertical axis indicates the correlation strength between each gene and shape. **(C, D)** Kyoto Encyclopedia of Genes and Genomes (KEGG) enrichment analysis of the 20 most highly expressed genes showing the relationship between the tan module and SP content. **(E)** Co-expression network plot of key genes of the tan module, including the top 100 genes in terms of connectivity. **(F)** Heatmap of core transcription factors of the tan module.

Our analysis of key genes related to SP content showed that the tan, green-yellow, and pink modules were correlated with SP content under cold stress, with the tan module showing the highest significance level. Within that module, we identified 119 differentially expressed hub genes (DEHGs; MM > 0.8, GS > 0.7) (**Figure 8B**), whose expression levels were upregulated in the M2 and M3 treatments and downregulated in the CK and M4 treatments (**Figure 8A**). According to GO analysis (**Figure 8D**), these key genes were enriched in the beta-fructo-furanosidase, hydrolase, and glycosyl bonding activity; *O*-glycosyl compound hydrolysis; and cellular carbohydrate metabolism pathways. KEGG pathway analysis showed that these DEHGs were enriched in the phenylpropane, secondary metabolite, flavonoid biosynthesis, riboflavin, and other metabolic pathways (**Figure 8C**). A co-expression network map of DEHGs in the tan module showed that it was closely related to SP content (**Figure 8E**). Transcription factor genes with high connectivity included *MYB20*, *WRKY53*, and *WRKY30*, all of which were upregulated with decreasing temperature; their highest expression levels were detected in the M2 treatment (**Figure 8F**).

### 3.9 Analysis of key genes associated with SOD accumulation

**Figure 9:**
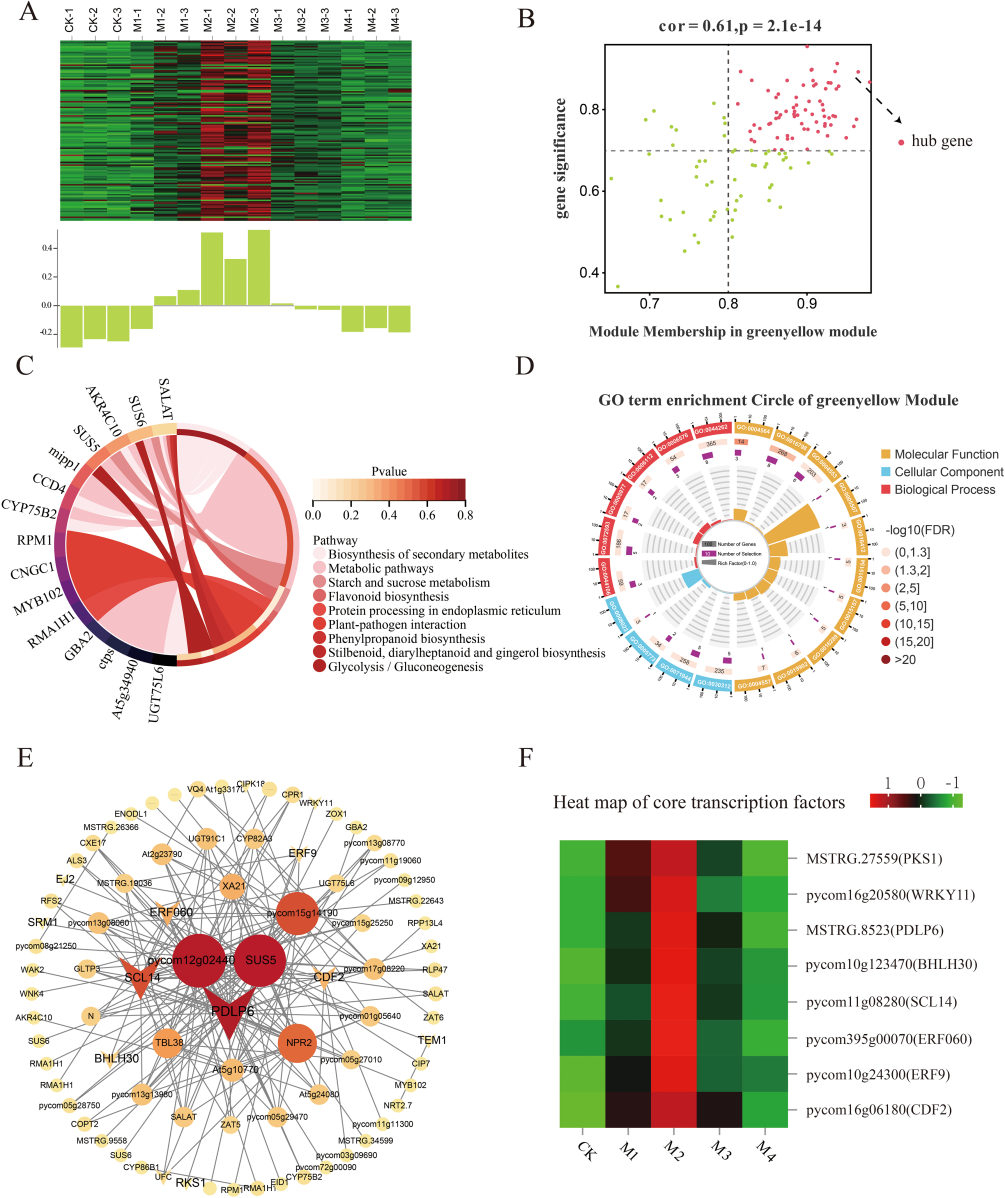
Co-expression network of the green-yellow module. **(A)** Gene co-expression heatmap of the green-yellow module (top) and the corresponding characteristic gene expression in each sample (bottom). **(B)** Plot of key genes with high expression and connectivity showing the relationship between superoxide dismutase (SOD) activity and the green-yellow module; the horizontal axis indicates the correlation strength between each expressed gene and a module, and the vertical axis indicates the correlation strength between each gene and shape. **(C)** Circle plot of Kyoto Encyclopedia of Genes and Genomes (KEGG) enrichment analysis results and **(D)** Gene Ontology (GO) enrichment plot of key gene enrichment rankings in the green-yellow module vs. SOD activity. **(E)** Co-expression network diagram of hub genes in the green-yellow module showing the connectivity of the top 100 key genes. **(F)** Heatmap of core transcription factors in the green-yellow module.

The key genes within the green-yellow, pink, and cyan modules were positively associated with SOD activity in the low-temperature treatments; the strongest association was observed with the green-yellow module. In that module, 74 DEHGs were identified (MM > 0.8, GS > 0.7; **Figure 9B**); their expression was found to decrease in the CK, M3, and M4 treatments and increase in the M1 and M2 treatments (**Figure 9A**). GO analysis showed that these key genes were associated with hydrolase activity, glycosyl bonds, cellular carbohydrate metabolic processes, reactive oxygen species, cellular polysaccharide, and metabolic processes (**Figure 9D**).

KEGG pathway analysis showed that the main pathways enriched in these DEHGs were starch, sucrose metabolism, and flavonoid biosynthesis (**Figure 9C**). The co-expression network map showed that the green-yellow module was closely related to SOD activity (**Figure 9E**), with the highest connectivity obtained for the transcription factors *PDLP6*, *SCL14*, *CDF2*, and *ERF9*, whose expression increased with decreasing temperature (**Figure 9F**).

### 3.10 Analysis of key genes associated with POD accumulation

**Figure 10:**
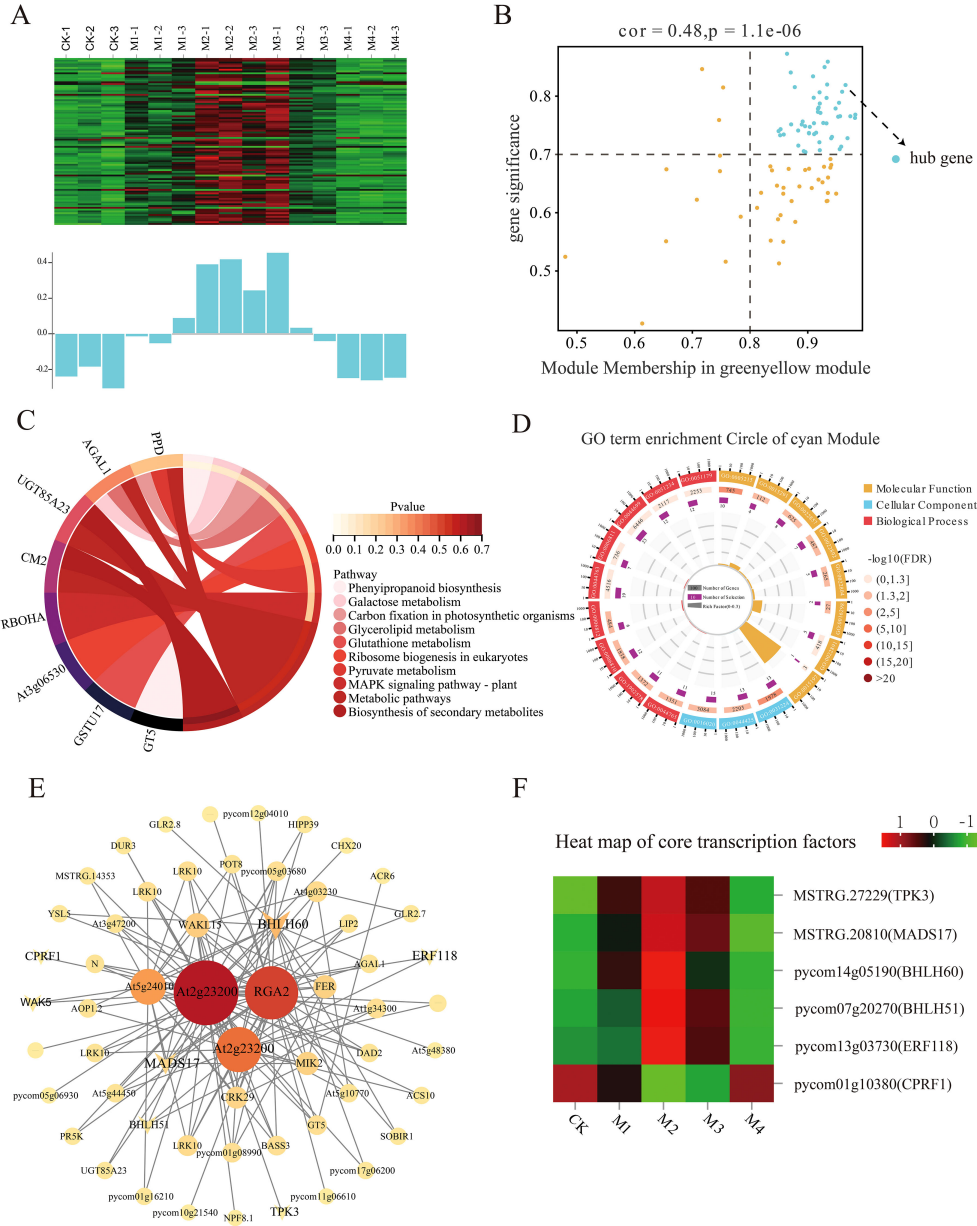
Co-expression network of the cyan module. **(A)** Gene co-expression heatmap of the cyan module (top) and the corresponding characteristic gene expression of each sample (bottom). **(B)** Dot plot of peroxidase (POD) activity and key genes in the cyan module; the horizontal axis shows the correlation strength between the expression of each gene and the module, and the vertical axis shows that between each gene and shape. Kyoto Encyclopedia of Genes and Genomes (KEGG) enrichment analysis results for **(C)** the cyan module and **(D)** POD activity. **(E)** Co-expression network plot of key genes of the cyan module showing the top 100 genes in terms of connectivity. **(F)** Heatmap of core transcription factors in the cyan module.

The cyan, green-yellow, and tan modules were significantly positively correlated with POD activity in the different temperature treatments. The most significant association was between the cyan module and 43 DEHGs (MM > 0.8, GS > 0.7; **Figure 10B**). The expression levels of these DEHGs decreased in the CK, M1, and M4 treatments and increased in the M2 and M3 treatments (**Figure 10A**). GO analysis showed that these DEHGs were enriched in the transporter activity, intrinsic membrane components, membrane parts, and single-organism transport pathways (**Figure 10D**). KEGG pathway analysis showed that these DEHGs were enriched mainly in genes involved in phenylpropanoid and secondary metabolite biosynthesis, metabolic pathways, and carbon fixation in photosynthetic organisms (**Figure 10C**). The co-expression network map of cyan module genes showed that those with the highest connectivity were *bHLH60* and *MADS17* (**Figure 10E**), whose expression increased with decreasing temperature (**Figure 10F**).

**Figure 11:**
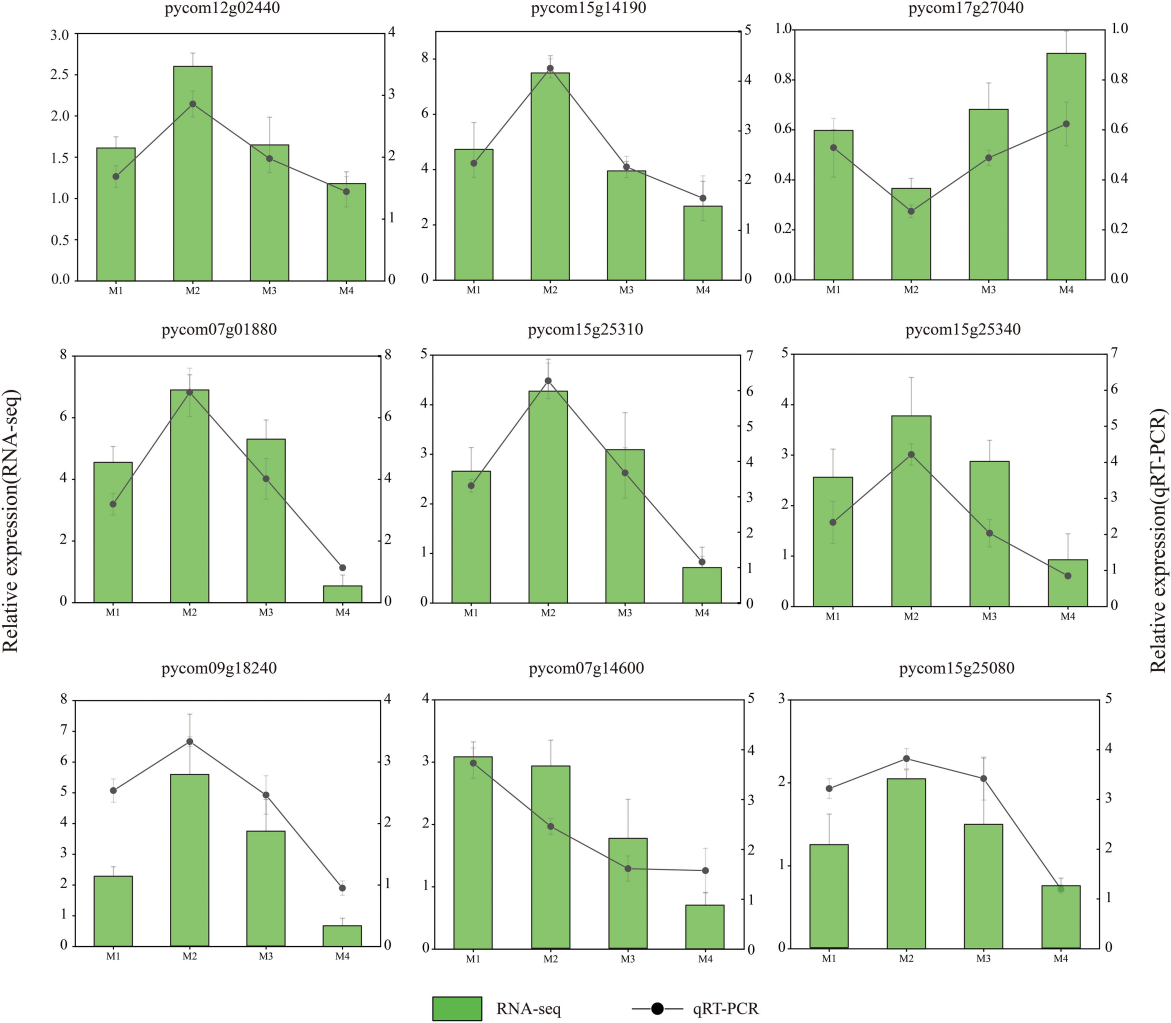
Validation of the RNA sequencing (RNA-seq) data by qRT-PCR assay. The column and black line represent the RNA-seq and qRT-PCR results, respectively.

To further verify the accuracy of the transcriptome results, six genes related to cold stress were selected from the abovementioned modules for the qRT-PCR assay. The results show that the expression level of the selected genes was largely consistent with the Fragments Per Kilobase of exon model per Million mapped fragments (FPKM) values (**Figure 11**), indicating the good reliability of the RNA-seq data.

## 4 Discussion

### 4.1 Pear fruit and flowers resist cold stress by increasing the activity of protective enzymes

Low-temperature stress strongly impacts the phenotypes of plants. In this study, the most obvious manifestations of cold damage on pear fruit were surface depression wilting and brown discoloration of the surface and flesh. Pear browning under low temperatures may be caused by high lysine and tryptophan content, high free radical activity, and oxidation due to increased polyphenol content (Lwin et al., 2023). Our KEGG analysis of pear fruit and flower organs showed enriched biosynthesis of stilbenes, diarylheptanoids, and gingerols, as well as enrichment in the phenylalanine, tyrosine, and tryptophan biosynthesis pathways. These results indicate the accumulation of tryptophan and phenolic compounds associated with browning fruit. At low temperatures, plant membrane systems are the primary sites of damage. The plasma membrane is the interface between the cell interior and the environment, and its stability is important for cold resistance; however, it is also the initial site of low-temperature stress damage (Zhao et al., 2018). Previous studies have shown that plant membrane systems with greater stability under cold stress are associated with higher cold resistance and lower relative conductivity. Changes in relative conductivity indicate changes in cell membrane permeability (Rosisca et al., 2019). MDA is a peroxidation product of the membrane system and a marker of the degree of membrane damage, including that induced by low temperature (Gonzalez et al., 2019). In this study, both the relative conductivity and MDA content of pear fruit and flower organs increased as the temperature decreased, which is consistent with the findings of previous studies.

Osmotic substances in plant cells confer cold resistance by effectively maintaining osmotic pressure in cold environments and reducing the freezing point (Li et al., 2013). In this study, KEGG analysis of DEGs between fruit and flower organs identified starch and sucrose metabolism, pentose and glucuronic acid conversion, and arginine and proline metabolism as the main pathways involved in the response to low-temperature stress. Most of the genes related to SP, Pro, and SS contents were upregulated. SOD, POD, and CAT are protective enzymes in the membrane lipid peroxidase-catalyzed defense system of plants. POD and CAT degrade excess H_2_O_2_ to reduce cellular damage that occurs under low-temperature stress (Garratt et al., 2002). Our results showed that as the temperature decreased, SOD and POD activities increased and then decreased. Transcriptome analysis identified the oxidoreductase pathway in fruit and flower organs as responsive to low-temperature stress, as demonstrated by the significant upregulation of most POD-related genes.

### 4.2 Pear fruit and flower organs resist cold stress by enhancing the expression of flavonoid-related genes

Flavonoids are important secondary metabolites that help plants resist diseases, pests, and low-temperature stress (Jiu et al., 2021). They also promote the formation of flower and fruit colors. In tomato plants, *SlMYB12* is a crucial regulator that promotes the biosynthesis of flavonoids by activating the expression of key enzymes like CHS, CHI, F3H, and FLS1 (Zhang et al., 2015). In this study, flavonoids in pear fruit and flower organs were found to be significantly responsive to low temperature, with significant changes in the expression of *CHS*, *HCT*, *ANS*, and *CHI* genes. *CHS*, the first key enzyme in the flavonoid biosynthetic pathway, catalyzes the synthesis of chalcone from *p*-coumaroyl-CoA (Tanaka et al., 2008). The expression levels of the three *CHS* genes changed significantly under cold stress, reaching their maximum in the M2 treatment in flower organs and the M1 treatment in fruit. *CHI* isomerization produces naringin, a colorless metabolite that enters other flavonoid synthesis pathways. In this study, *CHI* gene expression was the highest in flower organs in the M2 treatment and fruit in the M1 treatment. *HCT* catalyzes the hydroxylation of *p*-coumaroyl-CoA to caffeoyl-CoA. In flower organs, the expression levels of four *HCT* genes changed significantly in response to low temperature and were the highest in the M1 and M2 treatments, whereas in fruit, the expression levels of seven *HCT* genes changed significantly, with maximum values detected in the M1 treatment in four of these genes. These results are consistent with the beneficial role of flavonoids in fruit and flower organs.

### 4.3 Greater cold resistance in pear flower organs than in fruit

In pear trees, cold resistance varies depending on the growth period (Lee et al., 2023). In this study, the over-cooling point was found to be lower in flower organs than in fruit, with peak SOD and CAT activities and peak SS and Pro contents detected at lower temperatures (2.0°C lower) in flower organs than in fruit. POD and CAT activities and SS and SP contents were higher, whereas MDA content was lower, in flower organs than in fruit. Relative conductivity was lower in flower organs at colder temperatures than that of the M1 treatment, indicating that high levels of antioxidant enzyme activity and osmoregulatory capacity, together with low lipid peroxide levels, confer greater cold resistance in flower organs.

In the transcriptome, the total number of DEGs was higher in flower organs than in fruit. In addition, most of the upregulated DEGs in flower organs were detected in the M2 treatment, and most of those in fruit were detected in the M1 treatment. Genetic pathways common to fruit and flower organs were related to the phenylpropanoid and secondary metabolite biosynthesis and galactose metabolic pathways, although more DEGs in these pathways were detected in flower organs than in fruit. In flower organs, most genes in the shared flavonoid pathways were expressed in the M2 treatment, whereas in fruit, most were expressed in the M1 treatment. Similarly, most genes related to POD activity and SS and SP contents were expressed at lower temperatures in flower organs than in fruit, which may explain the greater cold sensitivity of fruit.

These results, together with the over-cooling point analysis results, may indicate that pear flower organs have greater cold resistance than pear young fruit. Pathways uniquely enriched in fruit included inorganic anion and cation transport, whereas those uniquely enriched in flower organs included pectic esterase activity, cell wall modification and organization, and phosphoester hydrolase activity. These differences in pathway enrichment may be the main factors accounting for the difference in cold sensitivity between pear fruit and flower organs.

### 4.4 WGCNA results for cold-responsive transcription factors

WGCNA was performed to construct gene regulatory networks to identify the key genes and physiological changes under cold stress in pear fruit. The results evaluated the association of SP content and SOD and POD activities with the module, with the strongest significant correlation observed between the tan module and SP content, the green-yellow module and SOD activity, and the cyan module and POD activity. The upregulated genes in these modules may reflect the pear fruit response to cold stress through membrane lipid peroxidase. The *R2R3-MYB* gene family encodes transcription factors that play important roles in plant growth and development as well as in stress resistance responses. In a previous study, *PhMYB62*, *GAUT12*, *CHS*, and *AUX 28* were shown to be involved in the regulation of cold stress in ‘Hebei’ pear flower organs (Li et al., 2024). The expression of *MYB20* in the tan module increased and then decreased as the temperature decreased, with the highest levels occurring in the M2 treatment. The *WRKY* gene family encodes plant-specific transcription factors that play important roles in abiotic stress (Wani et al., 2021; Song et al., 2018). The *PbWRKY31* gene is also involved in the regulation of cold sensitivity in pear plants, as its expression is sensitive to cold signals and the expression of other defense genes (Wei et al., 2023). In this study, the expression of *WRKY7*, *WRKY30*, *WRKY53*, *WRKY40*, and *WRKY11* in the green-yellow module increased with decreasing temperature. The *GRAS* gene family is widely involved in responses to abiotic stresses, including cold resistance responses, during plant growth. The expression level of the *GRAS* family member *SCL14*, identified in the green-yellow module, first increased and then decreased with decreasing temperature and was the highest in the M2 treatment, suggesting its importance in the pear fruit response to cold stress. The bHLH transcription factors also mediate plant stress responses. A genome-wide characterization of the *bHLH* gene in Chinese white pears showed that the expression of *PbrbHLH195* demonstrated its significant expression in relation to cold tolerance and decreased expression in relation to chlorophyll content, electrolyte leakage, and accumulation of MDA and H_2_O_2_ (Dong et al., 2021). *bHLH51* and *bHLH60* were identified in the cyan module; their expression first increased and then decreased with decreasing temperature. The expression of *bHLH93* in the cyan module decreased and then increased with decreasing temperature. The main transcription factors within the abovementioned three modules are summarized in **Supplementary Table S2**, all of which have been shown to participate in the defense responses to cold stress in other plant species, including *A. thaliana*, *Malus pumila*, and *Poncirus trifoliata*. Moreover, the upregulation of these genes in this study also suggests they may play crucial roles in pear cold resistance.

## 5 Conclusion

The study suggests that pear flower organs may exhibit greater cold resistance compared to young fruit. With decreasing temperatures, an increase in MDA and SS content was observed, alongside a fluctuating pattern in Pro and SP contents and the activities of CAT, SOD, and POD in both fruit and flower organs. Notably, the temperature thresholds at which these peaks occurred were lower in flowers, which could imply a higher degree of cold tolerance. Transcriptome analysis indicates that both fruit and flower organs potentially adapt to low temperatures by altering phenylpropane and flavonoid biosynthesis pathways, with transcription factors such as *MYB20*, *WRKY53*, *WRKY30*, *LDLP6*, *ERF9*, and *bHLH60* potentially playing significant roles in regulating these responses to cold stress. The observed higher antioxidant enzyme activity, osmoregulatory capacity, and lower lipid peroxidation in flower organs may be associated with their greater cold resistance; however, further research is needed to confirm these relationships.

## Data Availability

The raw data supporting the conclusions of this article will be made available by the authors, without undue reservation.
