# Supplementary material for: Integrated physiological and transcriptomic data revealed the cold-resistant mechanisms in reproductive organs of the ‘Jinguang’ pear cultivar
Source: Front Plant Sci. 2025 Feb 3;15:1501774. doi: 10.3389/fpls.2024.1501774 (PMC11830666; doi:10.3389/fpls.2024.1501774)
Supplement: Supplementary file 2 [file Table1.docx]

**Appendix A. Supplementary material**

**Analysis of physiological responses to cold stress and their molecular regulation in young fruit and flower organs of the ‘Jinguang’ pear cultivar**

Mengying Sun^a, b^, Shun Lin^a, b^, Zezhao Zhao^a^, Weizhen Guo^c^, Min Jiang^a, b^, Ying Li^c^, Jun Zhang^a, b, *^, Jingxian Zhao^c, *^, Minsheng Yang^a, b, *^

^a^ Hebei Agricultural University, Baoding, Hebei, 071000, China

^b^ Hebei Key Laboratory for Tree Genetic Resources and Forest Protection, Baoding, Hebei, 071000, China

^c^ Hebei Academic of Forestry and Grassland, Shijiazhuang, Hebei, 050061, China

^*^ Corresponding Authors:

E-mail address: zhangjunem@126.com (Jun Zhang); zhjx6902@163.com (Jingxian Zhao); yangms100@126.com (Minsheng Yang)

**This material includes:**

**Tables S1 and S2**

**Table S1 Primer information.**

| Gene ID | Primer sequence (5’-3’) |
| --- | --- |
| *tubulin* | F: CAAATGTGGGATGCCAAGAAC  R: CATCTGTTCGTCCACCTCTTT |
| *pycom12g02440* | F: CAAAGGAAACTCCAACGCCA  R: TCTCACCCTCCCCATACACG |
| *pycom15g14190* | F: TAAATACACGCTCACGCACG  R: TGATTAGACTGAAGCCCCAT |
| *pycom17g27040* | F: GACCCCAAATTCAACATCGC  R: TTCCCGTACCACTCAACCAA |
| *pycom15g25340* | F: CGAAGGCGAGCAAGAGTATC  R: CATCAAAAAGAGCAGCGGAG |
| *pycom07g01880* | F: GGCGGTGGAGAAGGTAGTAG  R: GTTTCGTCATCATTGGCAAA |
| *pycom15g25310* | F: CGCAGGAACGCTAAACATCT  R: GAACCGAACCAACCACAACA |
| *pycom09g18240* | F:AGCAAGAAGGAGAAGAAGAA |
|  | R:ACACCATCAATCAAGAACTC |
| *pycom07g14600* | F:ATGGCCAGAAGGACATACTTGG |
|  | R:TTAAGAGCGTGTGTTGTTGTGATTG |
| *pycom15g25080* | F:ATGGATTGCAGAGCCGTCG |
|  | R;CTAAAGGAGCTTGCTTGACGACG |

**Table S2 Response of the core transcription factors within different modules under chilling stress**

| Gene ID | TF | Module | Species | Gene transcriptional variation under chilling stress | Reference |
| --- | --- | --- | --- | --- | --- |
| *pycom09g18240* | *MYB20* | Tan | *Picea wilsonii* | *PmMYB20* expression was upregulated in *Picea wilsonii* at 4°C. | You et al., 2017 |
| *pycom15g25080* | *WRKY7* | Tan | *Hevea brasiliensis* | *HbWRKY7* and *HBWRKY11* expression levels were upregulated in *Hevea brasiliensis* in response to low temperature. | Zhai, 2013 |
| *pycom07g14630* | *WRKY30* | Tan | *Arabidopsis thaliana* | MRWRKY30 induced the expression of cold resistance genes such as *CBF* in *Arabidopsis thaliana*, and enhanced cold tolerance in transgenic *A*. *thaliana*. | Jiang et al., 2015 |
| *pycom01g22600* | *WRKY53* | Tan | *Oryza sativa* | OSWRKY53 negatively regulated cold tolerance at the booting stage in *Oryza sativa* | Fang et al., 2017 |
| *pycom08g04760* | *WRKY40* | Tan | *Malus pumila* | *MdWRKY40* overexpression promoted anthocyanin accumulation, blocked the repressor protein Mdmyb15L of Mdcbf2, and improved cold resistance in *Malus pumila*. | Xu et al., 2023 |
| *pycom10g24300* | *ERF9* | Greenyellow | *Poncirus trifoliata* | PTRERF9 enhanced cold resistance by positively regulating *PTRGSTU17* expression in *Poncirus trifoliata*, thereby promoting reactive oxygen species clearance under stress. | Zhang et al., 2022 |
| *pycom16g20580* | *WRKY11* | Greenyellow | *Brassica oleracea* | Low-temperature treatment of *WRKY11* induced significant upregulation in *Brassica oleracea.* | Chai et al., 2023 |
| *pycom11g08280* | *SCL14* | Greenyellow | *Juglans regia* | *JrGRAS5* expression was upregulated after 48 h of cold stress in *Juglans regia*; *JrGRAS51* and *SCL14* are closely related, suggesting mutual interaction of their proteins to improve cold resistance. | Liu et al., 2021 |
| *pycom07g20270* | *bHLH51* | Cyan | *Juglans regia* | *JrbHLH51* was significantly upregulated in *Juglans regia* after 48 h of cold stress. | Liu et al., 2023 |
| *pycom14g05190* | *bHLH60* | Cyan | *Prunus avium* | *PavbHLH60* was rapidly induced at low temperatures in *Prunus avium*, resulting in its upregulated expression. | Shen et al., 2021 |
| *pycom08g17980* | *bHLH93* | Blue | *Lepidium apetalum* | *bHLH93* expression was decreased in *Lepidium apetalum* seeds at low temperature. | Yuan et al., 2018 |
